# Supplementary material for: The “Dedicated” C.B.C.T. in Dentistry
Source: Int J Environ Res Public Health. 2023 May 25;20(11):5954. doi: 10.3390/ijerph20115954 (PMC10252385; doi:10.3390/ijerph20115954)
Supplement: Supplementary file 1 [file ijerph-20-05954-s001.zip › ijerph-2214883-supplementary/Suplementary .pdf]

Here there is an example of how it is possible today to carry out, with the necessary skills, volumetric examinations (cbct) with an acceptable diagnostic quality and an absolutely contained limited irradiation.

The examination in annex cone beam computed tomography (cbct) highlights all the diagnostic information necessary for correct treatment planning, and data relating to exposure and radiation factors, expressed directly by the cbct machine in d.a.p. (Dose area product).

This d.a.p. is particularly low even compared to a traditional two-dimensional examination, orthopantomography (opt). In fact in the annex, below, we have an opt with a dap of 114 mGy cm2 (given on the top left of the image opt) and a cbct dedicated and optimized for diagnostic purposes with 74 mGy cm2. With a lower absorbed dose we get better and more diagnostic information.

OPT

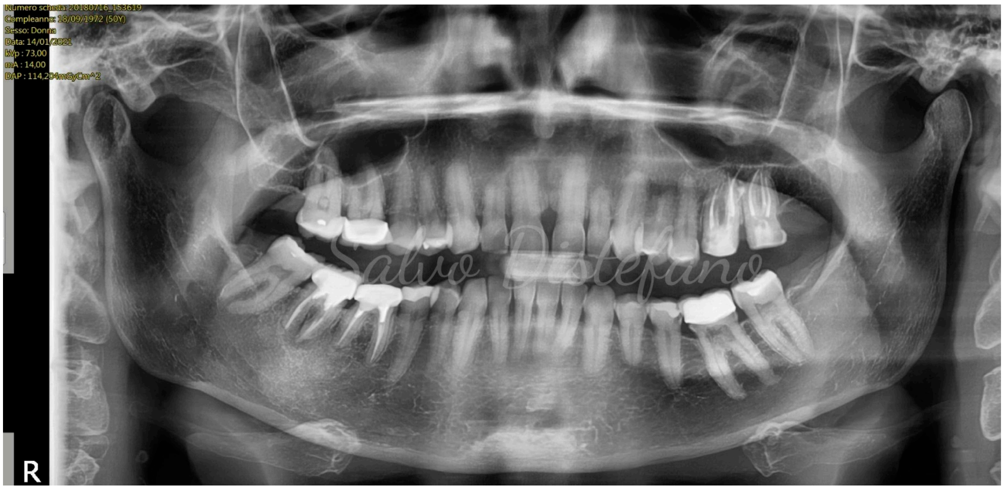

Small endodontic anatomical structures, such as The presence of a 'forgotten'' channel in the distal root of the first lower molar, are evident in the axial and sagittal plane, in the images below (cbct) despite the low dose from volumetric examination. (not visible with traditional 2d exams).

In addition, the image quality is acceptable, compared to the dose and the diagnostic task, despite the presence of metals (implant) and channeling filling, which usually determine image artifacts.

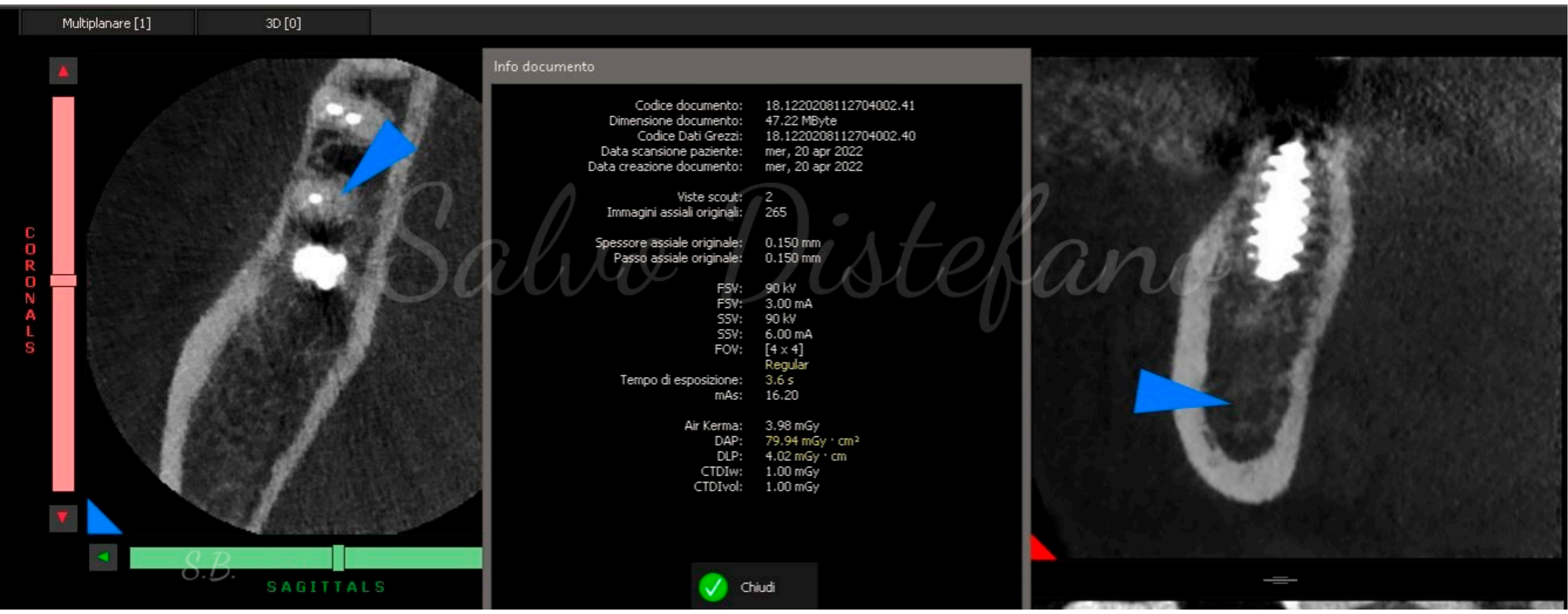
Multiplanare [1]
3D [0]
CORONALS
SAGITTALS
S.B.
Info documento
Codice documento: 18.1220208112704002.41
Dimensione documento: 47.22 MByte
Codice Dati Grezzi: 18.1220208112704002.40
Data scansione paziente: mer, 20 apr 2022
Data creazione documento: mer, 20 apr 2022
Viste scout: 2
Immagini assiali originali: 265
Spessore assiale originale: 0.150 mm
Passo assiale originale: 0.150 mm
FSV: 90 kV
FSV: 3.00 mA
SSV: 90 kV
SSV: 6.00 mA
FOV: [4 x 4]
Regular
Tempo di esposizione: 3.6 s
mAs: 16.20
Air Kerma: 3.98 mGy
DAP: 79.94 mGy · cm²
DLP: 4.02 mGy · cm
CTDIw: 1.00 mGy
CTDIvol: 1.00 mGy
Chiudi
Salvo Distefano

CBCT

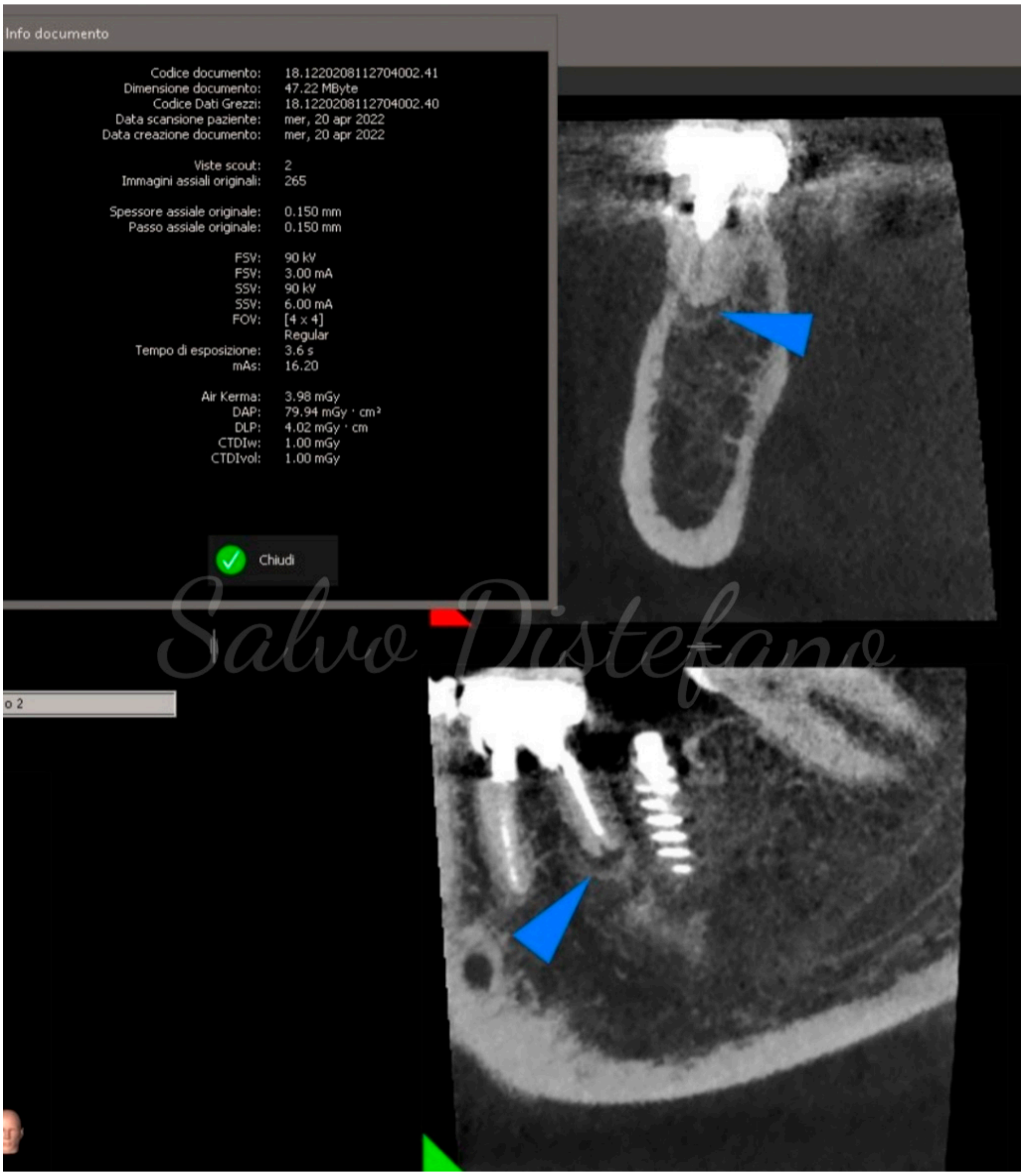
Info documento
Codice documento: 18.1220208112704002.41
Dimensione documento: 47.22 MByte
Codice Dati Grezzi: 18.1220208112704002.40
Data scansione paziente: mer, 20 apr 2022
Data creazione documento: mer, 20 apr 2022
Viste scout: 2
Immagini assiali originali: 265
Spessore assiale originale: 0.150 mm
Passo assiale originale: 0.150 mm
FSV: 90 kV
FSV: 3.00 mA
SSV: 90 kV
SSV: 6.00 mA
FOV: [4 x 4]
Regular
Tempo di esposizione: 3.6 s
mAs: 16.20
Air Kerma: 3.98 mGy
DAP: 79.94 mGy · cm²
DLP: 4.02 mGy · cm
CTDIw: 1.00 mGy
CTDIvol: 1.00 mGy
Chiudi
Salvo Distefano

CBCT
